# Evaluating the Accuracy and Explanatory Quality of Large Language Models ChatGPT, Claude, DeepSeek, Gemini, Grok, and Le Chat in Statistical Test Selection for Hypothesis Testing Decisions

**DOI:** 10.7759/cureus.94949

**Published:** 2025-10-19

**Authors:** Mukesh Shukla, Deepshikha Pandey, Samarjeet Kaur, Mayank Agarwal, Aayushi Goyal, Himanshi Sharma

**Affiliations:** 1 Community Medicine, All India Institute of Medical Sciences, Raebareli, Raebareli, IND; 2 Community Medicine, Amar Shaheed Jodha Singh Atayiya Thakur Dariyao Singh Medical College, Fatehpur, IND; 3 Community Medicine, Ganesh Shankar Vidyarthi Memorial Medical College, Kanpur, IND; 4 Physiology, All India Institute of Medical Sciences, Raebareli, Raebareli, IND

**Keywords:** chatgpt, claude, deepseek, gemini, grok, hypothesis, large language models, le chat, statistical test

## Abstract

Background

Large language models (LLMs) are increasingly integrated into academic and professional research workflows, yet their capability to accurately select appropriate statistical tests for hypothesis testing remains underexplored. Incorrect statistical test selection can lead to invalid conclusions and compromise scientific validity, making this evaluation critical for determining the reliability of LLMs in research applications. The study objective was to evaluate and compare the accuracy of six prominent LLMs (ChatGPT, Claude, DeepSeek, Gemini, Grok, and Le Chat) in selecting appropriate statistical tests for various hypothesis testing scenarios.

Materials and methods

A comparative, cross-sectional evaluation was conducted using 20 standardized statistical testing scenarios. Each scenario was designed to cover 20 different hypothesis testing situations, including comparisons of means, proportions, non-parametric alternatives, paired versus independent samples, and correlation and regression analyses. All models were prompted with identical instructions and evaluated by five independent experts with profound knowledge in biostatistics. Responses were assessed for accuracy and rated on five domains (clarity and accessibility, identification of necessary assumptions, pedagogical value, problem-solving approach, and statistical reasoning) using a five-point Likert scale. Analysis of Variance (ANOVA) was applied for between-group comparisons, and a p<0.05 was considered significant.

Results

All six LLMs achieved 100% accuracy in statistical test selection across all 20 hypothesis scenarios. However, significant variations emerged in explanatory quality. Claude demonstrated superior performance in clarity and accessibility (4.65 ± 0.58, p=0.05), while the problem-solving approach showed the most consistent excellence across models. Statistical reasoning exhibited variation ranging from 3.16 to 4.66, with complex regression methods receiving lower ratings than basic statistical tests. Gemini excelled in pedagogical value (4.50 ± 0.68), while ChatGPT ranked lowest in statistical reasoning despite strong problem-solving capabilities.

Conclusions

All LLMs demonstrate perfect accuracy in statistical test selection; however, differences exist in the quality of explanations and reasoning provided. These findings suggest that current-generation LLMs have become dependable tools for statistical consultation in hypothesis testing scenarios. However, users should consider model-specific strengths when seeking detailed explanations or educational content.

## Introduction

The increasing accessibility and capability of large language models (LLMs) have sparked significant interest in their potential to assist with academic and professional tasks, including statistical analysis [1]. As these models become integrated into educational tools, research workflows, and decision-making processes, their capacity to accurately select appropriate statistical tests for hypothesis-driven inquiries is especially important. Choosing the correct statistical test is a vital part of quantitative research, requiring an understanding of data characteristics, variable types, and underlying assumptions [2,3]. Errors in this stage can lead to invalid conclusions, misinterpretation of results, and broader implications for scientific validity [4].

LLMs have been found to exhibit increasingly natural language reasoning and domain-specific capabilities. However, empirical studies have not fully explored their comparative performance in applied statistical reasoning. While prior evaluations of LLMs have primarily focused on general reasoning, programming ability, and factual accuracy, there is a need to explore how reliably these LLMs can perform context-sensitive tasks like statistical test selection, particularly in hypothesis testing scenarios [5,6].

The researchers focused on hypothesis testing because making an incorrect choice in this area can have serious consequences, and they sought to determine whether these AI tools could be effectively utilized in academic and professional research work. This study aimed to test whether LLMs can accurately select the appropriate statistical tests for various research hypotheses and scenarios.

## Materials and methods

Study design and ethical considerations

This study employed a comparative, cross-sectional evaluation of six prominent LLMs: ChatGPT (OpenAI, San Francisco, CA, USA), Claude (Anthropic PBC, San Francisco, CA, USA), DeepSeek (High‑Flyer, Hangzhou, China), Gemini (Google, Mountain View, CA, USA), Grok (X.AI Corp., San Francisco, CA, USA), and Le Chat (Mistral AI, Paris, France). The models were evaluated based on their proficiency in accurately suggesting statistical tests for a set of hypothesis testing scenarios created by the research team. Free versions of these LLMs were used on a single day (August 21, 2025). Ethical approval was not needed because the study did not involve any human participants.

Scenario construction

A total of 20 unique statistical testing standardized scenarios (Appendix 1) were reviewed and prepared by the five experts who have sound knowledge in biostatistics. Three of the experts have more than 10 years of teaching experience and work as faculty in various medical institutes of India. The two experts were postgraduate trainees in the institute of national importance. The scenarios encompassed a spectrum of commonly tested hypotheses, with considerations for small sample sizes and underlying assumptions of normality and distribution. These included comparisons of means, comparisons of proportions, non-parametric alternatives, paired versus independent samples, and correlation and regression analysis. Each scenario was formulated in English, emulating the type of inquiry typically posed by researchers. Scenarios were designed to avoid ambiguity while requiring models to interpret key data characteristics such as variable types (categorical vs. continuous), group structure, and sample size.

Model prompting protocol

All models were prompted with identical instructions: "Given the following scenario, which statistical test should be used for hypothesis testing? Provide the name of the test and a brief rationale." Models were evaluated using their publicly accessible interfaces in August 2025. Each model was presented with the complete set of scenarios in randomized order to avoid prompt fatigue and bias.

Evaluation criteria

Model responses were independently evaluated by five users with profound knowledge in biostatistics. For accuracy, each response was categorized as correct (the test selected matches the statistically appropriate choice for the scenario) or incorrect (the test is statistically inappropriate given the scenario). The responses of these LLMs were assessed for clarity and accessibility, identification of necessary assumptions, pedagogical value, and problem-solving approach, and evaluated on a five-point Likert scale. Each response is assigned a numerical value (1 for strongly disagree to 5 for strongly agree), allowing for easy quantitative analysis [7].

Data analysis

The accuracy of each model was presented as the percentage of correctly identified statistical tests across all scenarios. Mean accuracy scores with standard deviations (SD) were computed to describe model performance. Descriptive statistics and between-group comparisons were performed using IBM SPSS Statistics for Windows (version 27.0; released 2020; IBM Corp., Armonk, NY) with analysis of variance (ANOVA) to test for significant differences. Statistical significance was set at p < 0.05 for all analyses.

## Results

Table 1 showed a remarkable finding regarding the accuracy of LLMs in selecting appropriate statistical tests for hypothesis testing. All six LLMs evaluated achieved a perfect 100% accuracy across all 20 statistical tests examined.

**Table 1:** Accuracy of LLMs in selecting the statistical test for hypothesis LLMs: large language models, ANOVA: analysis of variance

Statistical test for hypothesis testing	LLMs						
	ChatGPT	Claude	DeepSeek	Gemini	Grok		Le Chat
Student's t-test	Correct	Correct	Correct	Correct		Correct	Correct
One-way ANOVA	Correct	Correct	Correct	Correct		Correct	Correct
Mann-Whitney U test	Correct	Correct	Correct	Correct		Correct	Correct
Kruskal-Wallis H test	Correct	Correct	Correct	Correct		Correct	Correct
Fisher's exact test	Correct	Correct	Correct	Correct		Correct	Correct
Chi-square test	Correct	Correct	Correct	Correct		Correct	Correct
Log-rank test	Correct	Correct	Correct	Correct		Correct	Correct
Paired t-test	Correct	Correct	Correct	Correct		Correct	Correct
Repeated measures ANOVA	Correct	Correct	Correct	Correct		Correct	Correct
Wilcoxon signed-rank test	Correct	Correct	Correct	Correct		Correct	Correct
Friedman test	Correct	Correct	Correct	Correct		Correct	Correct
McNemar's test	Correct	Correct	Correct	Correct		Correct	Correct
Pearson's correlation	Correct	Correct	Correct	Correct		Correct	Correct
Spearman's correlation	Correct	Correct	Correct	Correct		Correct	Correct
Kappa agreement	Correct	Correct	Correct	Correct		Correct	Correct
Linear regression	Correct	Correct	Correct	Correct		Correct	Correct
Ordered logistic regression	Correct	Correct	Correct	Correct		Correct	Correct
Binary logistic regression	Correct	Correct	Correct	Correct		Correct	Correct
Multinomial logistic regression	Correct	Correct	Correct	Correct		Correct	Correct
Poisson regression	Correct	Correct	Correct	Correct		Correct	Correct
Total accuracy	100%	100%	100%	100%		100%	100%

Table 2 represents domain-specific analysis, revealing varying LLM strengths across five statistical performance areas. Clarity and accessibility scores were consistently high (4.00 to 4.83), with basic tests like Student's t-test performing better than correlation methods. The problem-solving approach showed the most consistent excellence (4.33 to 5.00). At the same time, statistical reasoning exhibited the highest variation (3.16 to 4.66), particularly struggling with complex regression methods like Poisson's regression.

**Table 2:** Pooled performance of LLMs in various domains in selecting a statistical test for hypothesis testing LLMs: large language models, ANOVA: analysis of variance

Test of significance for hypothesis	Domains of evaluation				
	Clarity and accessibility	Identification of necessary assumptions	Pedagogical value	Problem-solving approach	Statistical reasoning
Student's t-test	4.50 ± 0.54	4.66 ± 0.51	4.66 ± 0.51	5.00 ± 0.00	4.66 ± 0.81
One-way ANOVA	4.66 ± 0.51	4.33 ± 0.81	4.66 ± 0.51	5.00 ± 0.00	4.66 ± 0.51
Mann-Whitney U test	4.66 ± 0.51	4.66 ± 0.51	4.16 ± 0.75	4.83 ± 0.40	4.33 ± 0.81
Kruskal-Wallis H test	4.83 ± 0.40	4.16 ± 0.75	4.33 ± 0.51	4.83 ± 0.40	4.16 ± 0.40
Fisher's exact test	4.00 ± 0.00	4.50 ± 0.54	4.66 ± 0.51	4.33 ± 0.81	4.50 ± 0.54
Chi-square test	4.50 ± 0.54	4.50 ± 0.54	4.50 ± 0.54	4.50 ± 0.54	4.16 ± 0.75
Log-rank test	4.50 ± 0.54	4.66 ± 0.51	4.66 ± 0.51	4.33 ± 0.51	4.66 ± 0.51
Paired t-test	4.66 ± 0.51	4.33 ± 0.81	4.50 ± 0.83	5.00 ± 0.00	3.83 ± 0.98
Repeated measures ANOVA	4.33 ± 0.81	4.16 ± 0.40	4.50 ± 0.54	4.33 ± 0.81	3.50 ± 0.83
Wilcoxon signed-rank test	4.66 ± 0.51	4.66 ± 0.51	4.33 ± 0.81	4.66 ± 0.51	4.50 ± 0.83
Friedman test	4.16 ± 0.40	4.00 ± 0.00	4.66 ± 0.51	4.16 ± 0.75	4.16 ± 0.40
McNemar's test	4.16 ± 0.40	4.16 ± 0.40	4.33 ± 0.51	4.66 ± 0.51	4.16 ± 0.98
Pearson's correlation	4.50 ± 0.54	4.33 ± 0.81	4.16 ± 0.40	4.00 ± 0.63	3.33 ± 0.81
Spearman's correlation	4.00 ± 0.63	4.66 ± 0.51	4.16 ± 0.75	4.66 ± 0.51	3.50 ± 0.83
Kappa agreement	4.66 ± 0.51	4.50 ± 0.83	4.66 ± 0.51	4.50 ± 0.54	4.33 ± 1.03
Linear regression	4.00 ± 0.89	5.00 ± 0.00	3.66 ± 0.51	4.00 ± 0.89	4.33 ± 1.03
Ordered logistic regression	4.50 ± 0.54	3.00 ± 0.00	4.50 ± 0.54	4.66 ± 0.51	4.00 ± 1.09
Binary logistic regression	4.00 ± 0.89	3.83 ± 0.40	4.00 ± 0.63	3.66 ± 0.81	3.66 ± 1.03
Multinomial logistic regression	4.66 ± 0.51	3.66 ± 0.51	3.50 ± 0.54	3.50 ± 0.54	4.16 ± 0.40
Poisson regression	4.33 ± 1.03	3.33 ± 0.51	3.66 ± 0.51	3.33 ± 0.51	3.16 ± 0.40

Table 3 represents individual model comparisons, showing Claude leading in clarity and accessibility (4.65 ± 0.58) and matching Le Chat in statistical reasoning (4.20). At the same time, Gemini excelled in pedagogical value (4.50 ± 0.68). Only clarity and accessibility demonstrated statistically significant inter-model differences (p = 0.05), with other domains demonstrating non-significant variations despite observable performance differences. These findings suggest that while all LLMs achieve perfect test selection accuracy, their explanatory capabilities and reasoning quality vary considerably across statistical methods and models, with simpler tests generally receiving better ratings than advanced regression techniques.

**Table 3:** Domain-wise evaluation of individual LLMs selecting a statistical test for hypothesis testing LLMs: large language models

Domain	LLMs						
	ChatGPT	Claude	DeepSeek	Gemini	Grok	Le Chat	p-value
Clarity and accessibility	4.45 ± 0.60	4.65 ± 0.58	4.50 ± 0.7	4.25 ± 0.71	4.10 ± 0.44	4.60 ± 0.59	0.05
Identification of necessary assumptions	4.10 ± 0.71	4.50 ± 0.68	4.35 ± 0.67	4.30 ± 0.73	4.10 ± 0.71	4.15 ± 0.81	0.42
Pedagogical value	4.25 ± 0.78	4.40 ± 0.59	4.15 ± 0.74	4.50 ± 0.68	4.40 ± 0.59	4.20 ± 0.52	0.51
Problem-solving approach	4.60 ± 0.50	4.30 ± 0.80	4.20 ± 0.76	4.40 ± 0.82	4.60 ± 0.59	4.30 ± 0.73	0.36
Statistical reasoning	3.75 ± 0.78	4.20 ± 0.89	4.15 ± 0.87	4.05 ± 0.94	4.05 ± 0.88	4.20 ± 0.95	0.60

## Discussion

In the present study, the most striking observation was the exceptional performance of all six models, which achieved 100% accuracy across 20 diverse statistical test scenarios. This result contrasts with earlier studies, where researchers reported over 75% agreement between LLM-generated recommendations and appropriate statistical test choices, with acceptance rates generally exceeding 95% [8]. The observed discrepancy may suggest that the current generation of LLMs has undergone substantial improvements in statistical reasoning or that our evaluation framework assessed different facets of statistical competence compared with prior investigations.

Previous analyses demonstrated that models such as GPT-4 and Bing Chat correctly answered over 80% of statistical test selection questions, yet still fell short of perfect accuracy, leaving a margin of over 15% [9]. Our findings, therefore, align broadly with the trajectory of performance improvements reported in prior evaluations of LLMs in education and clinical reasoning domains. Furthermore, earlier research has shown that ChatGPT performed strongly in standardized medical licensing examinations, particularly for structured, knowledge-driven items [10]. Such evidence highlights the expanding role of LLMs in supporting decision-making and knowledge synthesis across specialized fields.

The domain-specific analysis in our study extends this body of work by revealing that although all six models demonstrated flawless accuracy in statistical test selection, they differed substantially in their capacity to justify these choices. This aligns with earlier research emphasizing that while LLMs demonstrate notable mathematical and logical capabilities, the depth, clarity, and pedagogical value of their explanations vary considerably [11]. By incorporating explanatory quality into the evaluation, our study contributes a novel dimension to understanding the educational usefulness of LLMs beyond correctness alone.

To the best of our knowledge, only one previous study has evaluated all six models simultaneously for accuracy and reliability, but in a different domain, blood physiology multiple-choice questions. That study found Claude to be the best-performing model. However, variation in performance across models was observed depending on the type of questions. This finding resonates with our observations in statistical reasoning [12].

Analysis of individual models showed that Claude consistently performed better, especially in clarity and ease of understanding. Although all models were equally accurate in selecting tests, their ability to explain statistical concepts varied. ChatGPT and Grok performed best in problem-solving, but ChatGPT ranked lowest in statistical reasoning. This showed a key difference: some models can correctly choose the proper test but struggle to explain the reasoning behind it. Such differences are significant for both teaching and professional use.

Limitations

The study has a few limitations. Firstly, the testing was conducted on a single day (August 21, 2025), providing a snapshot of model performance at that time. Since LLMs are updated frequently, their capabilities may evolve, underscoring the importance of ongoing evaluations to monitor improvements or declines. Additionally, this study was limited to free versions of these LLMs; premium or subscription versions may differ in performance, potentially offering greater accuracy and reliability. Furthermore, the scenarios are based upon the usual biostatistical analysis/statistical test done in basic biomedical research rather than advanced-level biostatistics.

## Conclusions

The analysis showed that while all LLMs demonstrate 100% accuracy in statistical test selection, there are notable differences in the quality of their explanations and reasoning, which mandate human overview and independent cross-checks. Claude consistently performed well across various domains, especially in clarity and statistical reasoning. The high performance across all models and domains suggested that LLMs have become dependable tools for statistical consultation. However, users should be aware that the quality of explanations and educational value may vary depending on the specific statistical method and the model selected.

## Appendices

Standardized hypothesis testing scenarios

1.     A physiologist wanted to study the effect of Drug X on serum cholesterol levels. He selected 120 individuals and randomly divided them into two groups of 60. Group A took Drug X daily for two months, while Group B did not. After two months, the mean serum cholesterol levels of both groups were compared.

2.     A researcher wanted to compare the mean haemoglobin levels in three groups, namely vegetarians, non-vegetarians, and mixed-diet individuals, each consisting of 100 randomly selected lactating females.

3.     A clinician wanted to compare the median duration of hospital stay between patients receiving oral and IV antibiotics.

4.     A researcher studied the median systolic blood pressure in four occupational groups (teachers, factory workers, farmers, and office clerks). The data were skewed.

5.     In a pilot study, a researcher examined the association between vaccination and disease occurrence in a small sample of 25 individuals, when the expected frequency in some cells was less than 5.

6.     In a particular study, the researchers aimed to explore the association between scabies infestation and residence in urban, rural, and slum areas.

7.     A trial aimed to study the survival rates among lung cancer patients receiving Drug A versus Drug B.

8.     In a study among 100 pregnant females, variations in haemoglobin were observed before and after fortified pulses were given for three months.

9.     A researcher compared the mean fasting blood sugar at baseline, three months, and six months in the same group of diabetic patients.

10.   A psychiatrist measured patients' anxiety scores before and after mindfulness training. The data were non-normal.

11.   A nutritionist compared the BMI of the same children measured at ages 5, 10, 15, and 20 years. The data were skewed.

12.   A study aimed to assess the presence or absence of worm infestation before and after mass drug administration of albendazole tablets.

13.   A researcher evaluated the correlation between body mass index and serum cholesterol in adults. The data were found to be normally distributed.

14.   A researcher examined the correlation between serum endorphin levels and time spent on physical exercise. The data were found to be skewed in distribution.

15.   A study aimed to assess the agreement between two radiologists, who independently interpreted X-rays for the presence or absence of pneumonia.

16.   A researcher predicted systolic blood pressure based on age, weight, height, and amount of oil consumption in the diet.

17.   A study investigated factors predicting the severity of pain in arthritis patients with obesity (yes/no), smoking (yes/no), and exercise (yes/no).

18.   A researcher studied the effect of obesity (yes/no), smoking (yes/no), and exercise (yes/no) on the likelihood of developing hypertension (yes/no).

19.   A study investigated predictors of the type of delivery (normal vaginal, assisted vaginal, caesarean) among pregnant women, considering age, residence, history of abortion, and the health personnel who conducted the delivery.

20.   A study modelled the number of dengue cases reported per household based on breeding sites, sanitation level, and water storage practices.
